# Tetraarsenic Hexoxide Enhanced the Anticancer Effects of *Artemisia annua* L. Polyphenols by Inducing Autophagic Cell Death and Apoptosis in Oxalplatin-Resistant HCT116 Colorectal Cancer Cells

**DOI:** 10.3390/ijms26167661

**Published:** 2025-08-08

**Authors:** Eun Joo Jung, Hye Jung Kim, Sung Chul Shin, Gon Sup Kim, Jin-Myung Jung, Soon Chan Hong, Choong Won Kim, Won Sup Lee

**Affiliations:** 1 Department of Internal Medicine, Institute of Medical Science, Gyeongsang National University Hospital, Gyeongsang National University College of Medicine, 15 Jinju-daero 816 Beon-gil, Jinju 52727, Republic of Korea; eunjoojung@gnu.ac.kr; 2 Department of Pharmacology, Institute of Medical Science, Gyeongsang National University College of Medicine, Jinju 52727, Republic of Korea; 3 Department of Chemistry, Research Institute of Life Science, Gyeongsang National University, Jinju 52828, Republic of Korea; sshin@gnu.ac.kr; 4 Research Institute of Life Science, College of Veterinary Medicine, Gyeongsang National University, Jinju 52828, Republic of Korea; gonskim@gnu.ac.kr; 5 Department of Neurosurgery, Institute of Medical Science, Gyeongsang National University Hospital, Gyeongsang National University College of Medicine, Jinju 52727, Republic of Korea; gnuhjjm@gnu.ac.kr; 6 Department of Surgery, Institute of Medical Science, Gyeongsang National University Hospital, Gyeongsang National University College of Medicine, Jinju 52727, Republic of Korea; hongsc@gnu.ac.kr; 7 Department of Biochemistry, Institute of Medical Science, Gyeongsang National University College of Medicine, Jinju 52727, Republic of Korea; cwkim@gnu.ac.kr

**Keywords:** *Artemisia annua* L. polyphenols (pKAL), As_4_O_6_, γ-H2AX, caspase-8, cleaved form of p62, intracellular vesicles, oxaliplatin-resistant (OxPt-R), colorectal cancer, wortmannin, NAC

## Abstract

It was reported that polyphenols extracted from Korean *Artemisia annua* L. (pKAL) have higher anticancer effects in oxaliplatin-resistant (OxPt-R) HCT116 cells than in HCT116 cells. In this study, it was tested whether and how As_4_O_6_ enhances anticancer effects of pKAL in HCT116 and HCT116-OxPt-R colorectal cancer cells. The CCK-8 assay, phase-contrast microscopy, and colony formation assay revealed that As_4_O_6_ enhanced anticancer effects of pKAL, with induction of nuclear deformity and intracytoplasmic vesicle formation in both cells. Western blot analysis revealed that co-treatment with As_4_O_6_ and pKAL significantly decreased the expression of NF-kB, EGFR, cyclin D1, CD44, and β-catenin, and upregulated the expression of p62 and LC3B in both cells. It also induced the activation of caspase-8 and γ-H2AX and the cleavage of β-catenin, PARP1, lamin A/C, and p62. These phenomena were inhibited by wortmannin, and further suppressed by co-treatment of wortmannin with an ROS inhibitor, N-acetyl cysteine. This study suggests that As_4_O_6_ enhanced the anticancer effects of pKAL by inducing autophagic cell death accompanied by apoptosis in both parental HCT116 and HCT116-OxPt-R cells. It also suggests that ROS generation and the downregulation of AKT, NF-κB p65, cyclin D1, EGFR, and β-catenin may play an important role in the As_4_O_6_-enhanced anticancer effect of pKAL.

## 1. Introduction

Colorectal cancer is one of the most common cancers in men and women worldwide. Despite continuous development and improvement in cancer treatment, its mortality rate is still high (five-year survival rates around 10% to 28% for inoperable colorectal cancer) due to chemoresistance followed by aggravation of cancer progression. In colorectal cancer treatment, oxalipatin (OxPt)-based chemotherapy is widely used as first-line chemotherapy. It is used under the name FolFox chemotherapy for postoperative high-risk colorectal cancer or metastatic colorectal cancer [1]. Therefore, most recurrent or advanced colorectal cancer patients who have received chemotherapy are considered to have OxPt resistance, so overcoming OxPt resistance (OxPt-R) is a very important task. In addition, OxPt belongs to the group of platinum-containing anticancer compounds such as cisplatin and carboplatin [2] and is widely used in the treatment of various types of tumors originating in the digestive system such as gastric, pancreatic, biliary, and colorectal cancer.

The action mechanism of OxPt is that it intercalates between double-strand DNA and forms intrastrand linkages between two adjacent guanine residues or between guanine and adenine, thereby inducing DNA damage and reactive oxygen species (ROS), and inhibiting DNA replication and transcription [2,3]. The anticancer properties of OxPt are mainly associated with the induction of apoptosis, regulated necrosis, autophagy, and senescence [4]. The anticancer effects are associated with p53-dependent and p53-independent apoptotic cell death signaling under oxidative stress, which is generally suppressed by the ROS inhibitor N-acetyl cysteine (NAC) [2,3]. Therefore, OxPt resistance is associated with alteration of p53 function [5,6], increased inhibitors involved in apoptosis, and up- or downregulation of signaling to evade cell death mechanisms [4,7,8].

*Artemisia annua* L. (Gaddongsook, Korean), an annual herb, has been used as a Korean folk medicine for a long time [9]. Many kinds of compounds extracted from *A. annua* L. have exhibited chemotherapeutic effects and immune regulation [9,10]. Among them, quercetin, kaempferol, luteolin, isorhamnetin, artemisinin, and stigmasterol were identified as active components of *A. annua* L. through bioinformatic analysis [9,10]. pKAL compounds extracted from Korean *A. annua* L. are composed of well-known active polyphenols and unknown polyphenols [9,11], which consist of 17 polyphenols as described in Section 4. Our previous study demonstrated that polyphenols extracted from Korean *A. annua* L. (pKAL) exhibited anticancer effects by inhibiting the EMT process without significant cytotoxicity to normal cells [12] and by suppressing a stem cell phenotype and β-catenin in radio-resistant MDA-MB-231 human breast cancer cells [12,13]. In addition, we also found that the anticancer effects of pKAL on HCT 116 colorectal cancer cells have been attenuated by upregulation of well-known survival proteins such as NGF-β, VEGFD, and insulin [14]. Therefore, we sought to find a way to increase the anticancer effect of pKAL.

Arsenic compounds such as arsenic trioxide (As_2_O_3_) and tetraarsenic hexoxide (As_4_O_6_) have shown anticancer effects by inhibiting nuclear factor kappa B (NF-κB) signaling and activating apoptotic signaling in various cancer cells [15,16,17,18]. As_2_O_3_ was approved by the US Food and Drug Administration (FDA) and has been used in the treatment of acute promyelocytic leukemia and various solid tumors including liver cancer, pancreatic cancer, lung cancer, and colorectal cancer [19,20,21,22]. In addition, As_2_O_3_ reduces chemoresistance induced by 5-fluorouracil and cisplatin in HBx-HepG2 cells via suppression of drug resistance signaling and activation of apoptotic cell death signaling [23]. However, various toxicities in humans have been found in a second-line treatment of refractory metastatic colorectal cancer due to As_2_O_3_-based chemotherapy [22]. Therefore, further studies are needed to find more effective and safe chemotherapeutic strategies that can suppress various toxicities caused by As_2_O_3_-based chemotherapy until the day when we conquer cancer.

Tetraarsenic hexoxide (As_4_O_6_) has been used as a Korean folk remedy for the management of cancer since the late 1980s, and no serious toxicities have been observed. Previous studies have shown that the anticancer effects of As_4_O_6_ are more potent than those of As_2_O_3_ in human cancer cells in vitro, and that the signaling pathways of As_4_O_6_-induced cell death are different from those of As_2_O_3_ [18]. In addition, we also found out that As_4_O_6_ harbors anticancer properties by suppressing NF-κB activity or PI3K/Akt signaling that is related to antiapoptosis, as well as angiogenesis signaling [17,24]. In addition, we thought that a novel chemotherapeutic strategy combining different types of chemotherapeutic agents and natural products may have more effective and safer anticancer effects on drug-resistant cancer or increase anticancer effects.

Therefore, this study investigated whether the addition of As_4_O_6_ could address the phenomenon of the anticancer efficacy of pKAL decreasing over time in parental HCT116 cells [25]. In addition, this study investigated whether As_4_O_6_ could enhance the anticancer efficacy of pKAL in HCT116-OxPt-resistant (OxPt-R) cells that our team previously generated and characterized [25], and whether the mechanism was related to OxPt-R proteins. Therefore, this study was designed to compare the anticancer effects of pKAL with As_4_O_6_ in both parental HCT116 and HCT116-OxPt-R cells and to identify the mechanism.

## 2. Results

### 2.1. Anticancer Effect of As_4_O_6_ on the Survial of HCT116 and HCT116-OxPt-R Cells

**Figure 1:**
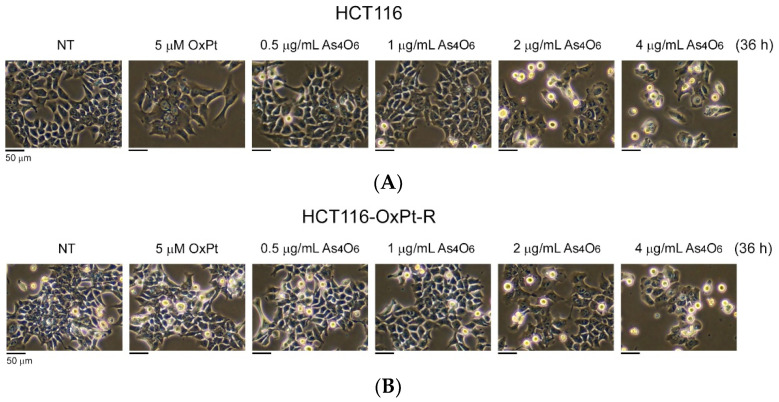
Effect of OxPt and As_4_O_6_ on cell morphology in HCT116 and HCT116-OxPt-R cells, respectively: (**A**,**B**) Cells were cultured in a 10 cm dish for 20 h, and then treated with the indicated concentrations of drugs for 36 h. Cell morphology was analyzed by phase-contrast microscopy.

To determine whether As_4_O_6_ exhibits an anticancer effect in HCT116-OxPt-R colorectal cancer cells exhibiting drug resistance to 5 μM OxPt, we first examined the morphological changes induced by As_4_O_6_ in parental HCT116 cells and HCT116-OxPt-R cells using phase-contrast microscopy. As expected, treatment with 5 μM OxPt for 36 h induced significant morphological changes in HCT116 cells, but not in HCT116-OxPt-R cells (Figure 1A,B, panels 1, 2). Treatment with 2 μg/mL and 4 μg/mL As_4_O_6_ for 36 h also induced significant morphological changes in both cells, whereas 0.5 μg/mL and 1 μg/mL As_4_O_6_ did not. In particular, the ability of As_4_O_6_ to induce morphological changes was found to be slightly lower in HCT116-OxPt-R cells than in HCT116 cells (Figure 1A,B, panels 3–6). These results suggest that the anticancer effect of As_4_O_6_ treatment on both cancer cells is insignificant at concentrations below 1 μg/mL As_4_O_6_ and induces similar degrees of morphological changes in both cell types.

### 2.2. Anticancer Effect of Combination Treatment of As_4_O_6_ and pKAL on HCT116-OxPt-R Cells

**Figure 2:**
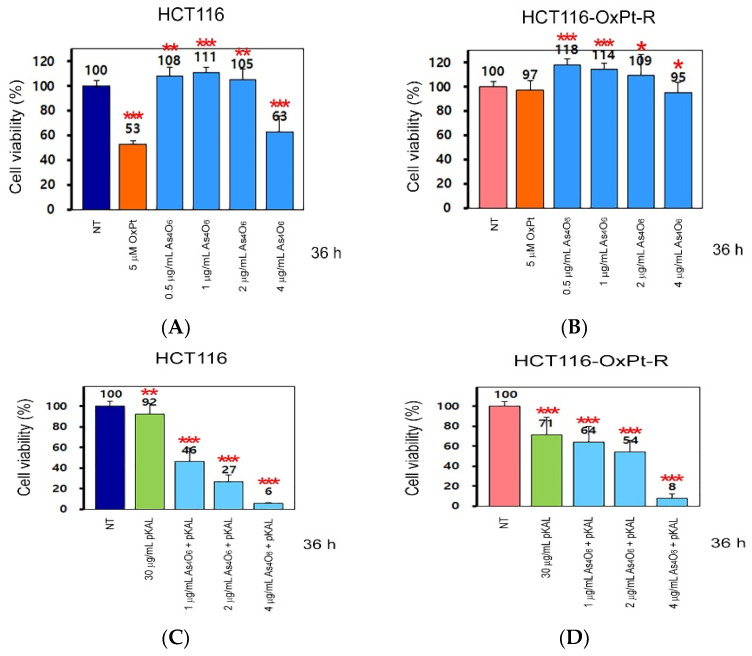
Regulation of cell viability by As_4_O_6_ and pKAL in HCT116 and HCT116-OxPt-R cells: (**A**–**D**) Cells were cultured in a 24-well plate for 20 h, treated with the indicated concentrations of drugs for 36 h, and then analyzed by the CCK-8 assay. Statistical significance was determined using Student’s *t*-test: * *p* < 0.05; ** *p* < 0.005; *** *p* < 0.001.

Next, to determine whether the anticancer effect of As_4_O_6_ could be enhanced by pKAL, we analyzed the cell viability regulated by As_4_O_6_ and pKAL in HCT116 and HCT116-OxPt-R cells using a cell counting kit-8 (CCK-8) reagent. Treatment with 5 μM OxPt for 36 h significantly reduced cell viability in HCT116 cells (53%), but not in HCT116-OxPt-R cells (97%) (Figure 2A,B, graph bars 1, 2). In addition, while cell viability was not significantly reduced in both cells when treated with 0.5 to 2 μg/mL As_4_O_6_ for 36 h, it was slightly reduced in HCT116-OxPt-R cells (95%) and more markedly in HCT116 cells (63%) when treated with 4 μg/mL As_4_O_6_ for 36 h (Figure 2A,B, graph bars 3–6). In particular, the inhibitory effect of 30 μg/mL pKAL on cell viability was higher in HCT116-OxPt-R cells (71%) than in HCT116 cells (92%), and it was further enhanced by As_4_O_6_ in a concentration-dependent manner in both cells: in HCT116 cells, 1 μg/mL As_4_O_6_ + pKAL (46%), 2 μg/mL As_4_O_6_ + pKAL (27%), and 4 μg/mL As_4_O_6_ + pKAL (6%); and in HCT116-OxPt-R cells, 1 μg/mL As_4_O_6_ + pKAL (64%), 2 μg/mL As_4_O_6_ + pKAL (54%), and 4 μg/mL As_4_O_6_ + pKAL (8%) (Figure 2C,D, graph bars 2–5). These results suggest that the effect of pKAL was higher in HCT116-OxPt-R cells and that the effect was enhanced. On the other hand, although the effect of pKAL was lower in HCT116 cells, the increase in anticancer efficacy was greater when they were co-treated with As_4_O_6_ than in HCT116-OxPt-R cells. These findings suggest that the cooperative anticancer effect of As_4_O_6_ and pKAL was observed in both cancer cells.

**Figure 3:**
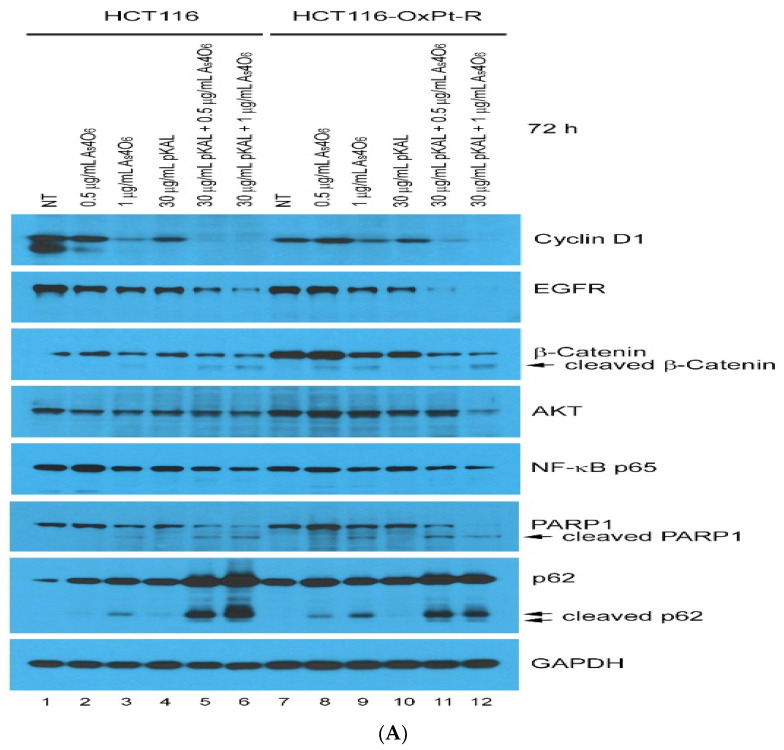

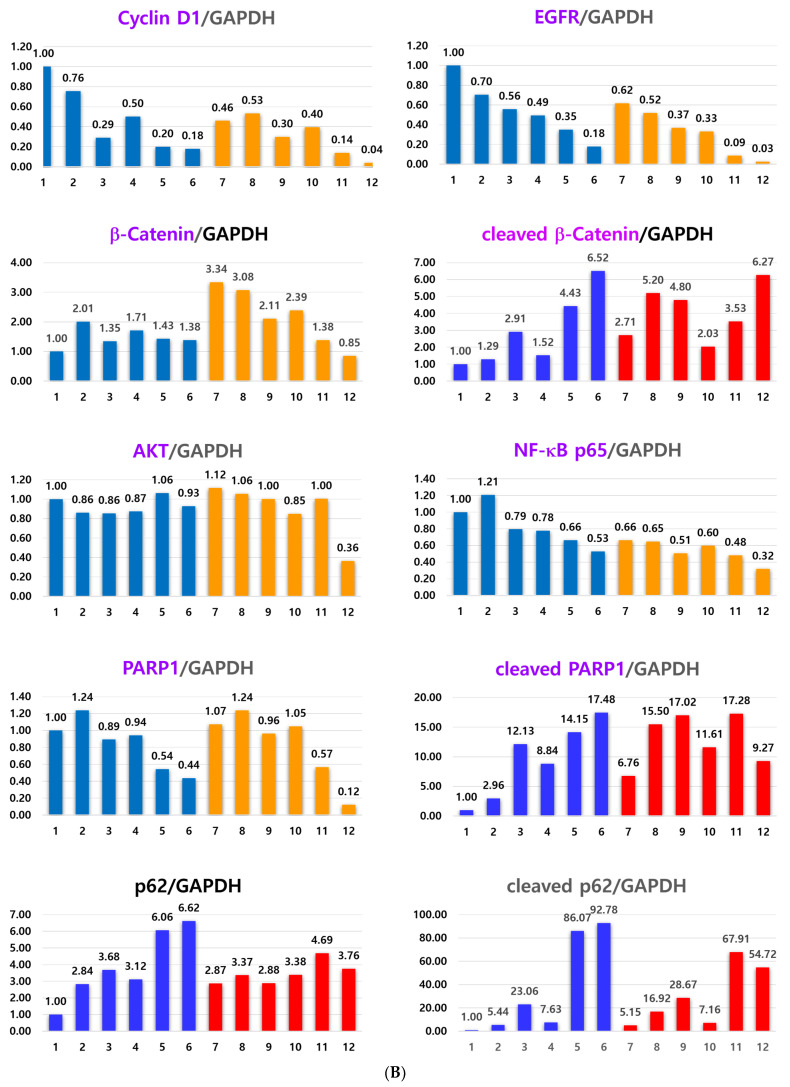
Regulation of protein levels by As_4_O_6_ and pKAL in HCT116 and HCT116-OxPt-R cells: (**A**) Cells were grown in a 10 cm culture dish for 20 h, and then treated with the indicated concentrations of drugs for 72 h. Whole-cell extracts were analyzed by Western blot using the indicated antibodies. (**B**) Densitometry analysis of protein bands in Figure 3A was conducted using the ImageJ program.

To elucidate the cooperative anticancer mechanism induced by combined treatment with pKAL and As_4_O_6_ in HCT116-OxPt-R cells, changes in proteins involved in the cell cycle, survival, and death signaling were investigated by Western blot and densitometry analysis. Western blot analysis revealed that combined treatment significantly decreased the expression of cyclin D1, EGFR, and β-catenin and increased the expression of p62, as shown in Figure 3. In addition, treatment with As_4_O_6_ alone led to p62 proteins being cleaved in a dose-dependent manner in both HCT116 and HCT116-OxPt-R cells (lanes 1–3 and 7–9). Moreover, the cleaved form of p62 and PARP1 was remarkably upregulated by the combined treatment, suggesting that the synergistic cytotoxicity may be related to inducing apoptosis, inflammation, or other cell death processes. As shown in Figure 3, the decrease in EGFR, β-catenin, NF-kB p65, AKT, and PARP1 required for cell survival was more significantly reduced in HCT116-OxPt-R cells by combined treatment than in HCT116 cells. These results suggest that the cooperative anticancer effect of the combination treatment with As_4_O_6_ and pKAL is exerted in both HCT116 and HCT116-OxPt-R cells through regulation of the cell cycle (cyclin D1), survival (EGFR, AKT, NF-κB p65), death (cleavage of PARP1 and p62), and drug resistance (β-catenin and p62). In addition, these results suggest that although the response to the action protein of the combination therapy is the same in both cell lines, the anticancer effect could be different because it is determined by how important the suppressed proteins are to the survival of the cancer cells.

### 2.3. Induction of Intracellular Vesicles by Combined Treatment of As_4_O_6_ and pKAL

**Figure 4:**
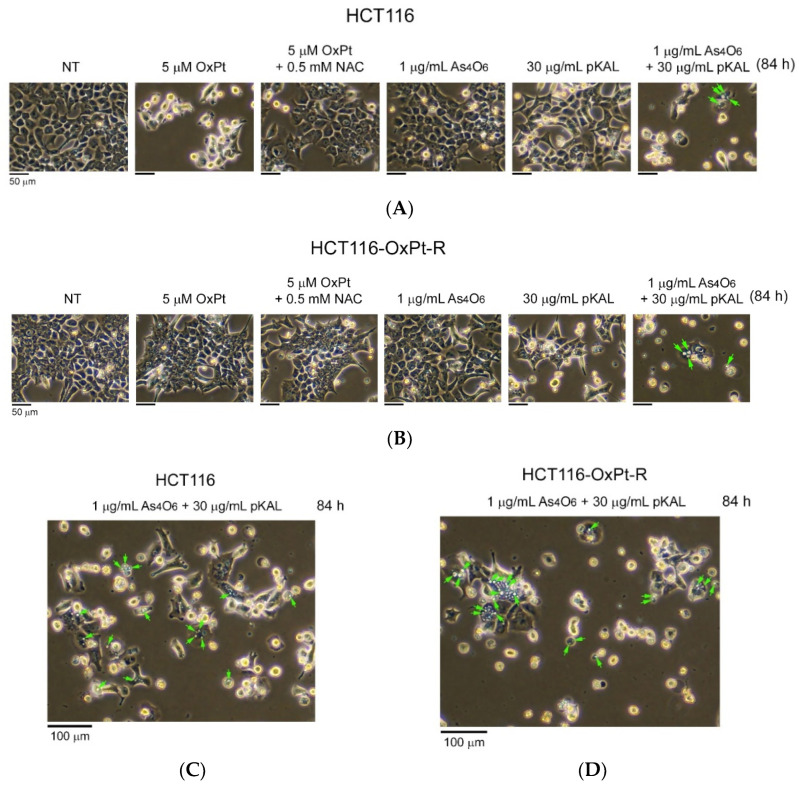
Long-term effects of As_4_O_6_ and pKAL on morphological changes in HCT116 and HCT116-OxPt-R cells: (**A**–**D**) Cells were cultured in a 6-well plate for 20 h, and then treated with the indicated concentrations of drugs for 84 h. Morphological changes in whole cells were analyzed by phase-contrast microscopy. Representative intracellular vesicles are indicated by arrows (**C**,**D**). The panels of Figure 4A,B are numbered starting from the left.

**Figure 5:**
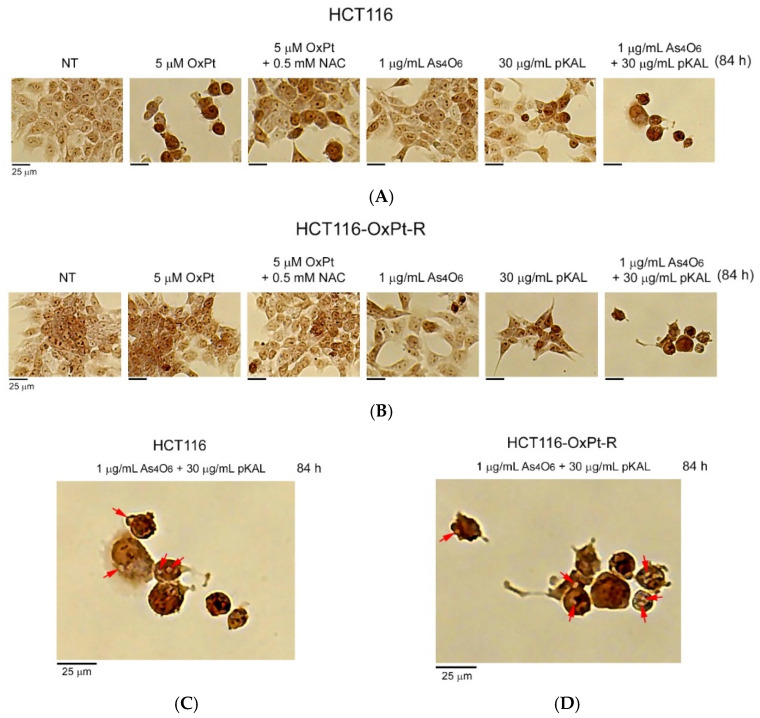
Long-term effects of As_4_O_6_ and pKAL on nuclear morphological changes in HCT116 and HCT116-OxPt-R cells: (**A**–**D**) Cells were grown for 20 h on a 6-well plate, and then treated with the indicated concentrations of drugs for 84 h. Nuclear staining of attached cells was analyzed by phase-contrast microscopy after hematoxylin staining. Representative intracellular vesicles in the Figure 5A,B panel 6 are indicated by arrows (**C**,**D**). The panels of Figure 5A,B are numbered starting from the left.

To further investigate the anticancer effect of combined treatment of As_4_O_6_ and pKAL in HCT116 and HCT116-OxPt-R cells, phase-contrast microscopy was used to examine the morphological changes in cells after long-term treatment for 84 h. As shown in Figure 4, long-term treatment with 5 μM OxPt for 84 h induced more significant morphological changes in HCT116 cells than did the 36 h treatment in Figure 1 (panels 1, 2), while OxPt did not induce significant changes in HCT116-OxPt-R cells (Figure 5B, panels 1, 2). The morphological changes induced by 5 μM OxPt in HCT116 cells were inhibited by the 0.5 mM NAC treatment, suggesting that ROS may play an important role in the anticancer effect of OxPt (Figure 4A, panels 2, 3). However, no significant morphological changes were observed in both cells when treated with 1 μg/mL As_4_O_6_ for 84 h (Figure 4A,B, panels 1, 4). The results in Figure 2 show that the cytotoxicity induced by 30 μg/mL pKAL was higher in HCT116-OxPt-R cells than in HCT116 cells, and this effect was markedly enhanced by co-treatment with 1 μg/mL As_4_O_6_ in both cells. Consistent with these results, 30 μg/mL pKAL significantly changed cell morphology in HCT116-OxPt-R cells compared with HCT116 cells, and this effect was further enhanced by co-treatment with 1 μg/mL As_4_O_6_ and 30 μg/mL pKAL for 84 h (Figure 4A, panels 1, 4–6). Furthermore, our results demonstrated that co-treatment with As_4_O_6_ and pKAL induced some intracellular vesicles in both cells, as representatively indicated by arrows (Figure 5C,D). These results suggest that the cooperative anticancer effect of As_4_O_6_ and pKAL is related to cellular morphological changes associated with the induction of intracellular vesicles in both HCT116 and HCT116-OxPt-R cells.

To further investigate the nuclear morphological changes induced by combined treatment with As_4_O_6_ and pKAL in HCT116 and HCT116-OxPt-R cells, hematoxylin staining was performed on attached cells after long-term treatment. Consistent with Figure 4A, treatment with 5 μM OxPt for 84 h significantly reduced the cell number in HCT116 cells, but not in HCT116-OxPt-R cells (Figure 5, panels 1, 2). The nuclear morphological changes induced by 5 μM OxPt in HCT116 cells were inhibited in the presence of 0.5 mM NAC (Figure 5A, panels 2, 3). However, in HCT116 cells, 1 μg/mL As_4_O_6_ treatment for 84 h did not cause significant nuclear morphological changes, whereas in HCT116-OxPt-R cells, the treatment resulted in changes in nuclear morphologies and cell number. This suggests that long-term treatment with 1 μg/mL As_4_O_6_ may result in a more potent anticancer effect in HCT116-OxPt-R cells than in HCT116 cells (Figure 5, panels 1, 4). Moreover, nuclear morphology was more significantly changed by 30 μg/mL pKAL in HCT116-OxPt-R cells than in HCT116 cells, and the changes were more remarkable when the cells were co-treated with 1 μg/mL As_4_O_6_ and 30 μg/mL pKAL for 84 h (Figure 5, panels 5, 6). In particular, our results revealed that long-term co-treatment with As_4_O_6_ and pKAL significantly induced nuclear structural changes and intracellular vesicles in both cells, which are representatively indicated by arrows (Figure 5C,D), compared to the 36 h co-treatment with As_4_O_6_ and pKAL (Figure 1). These results suggest that the cooperative anticancer effect of As_4_O_6_ and pKAL combination treatment is associated with the induction of nuclear structural changes and intracellular vesicles in both HCT116 and HCT116-OxPt-R cells.

**Figure 6:**
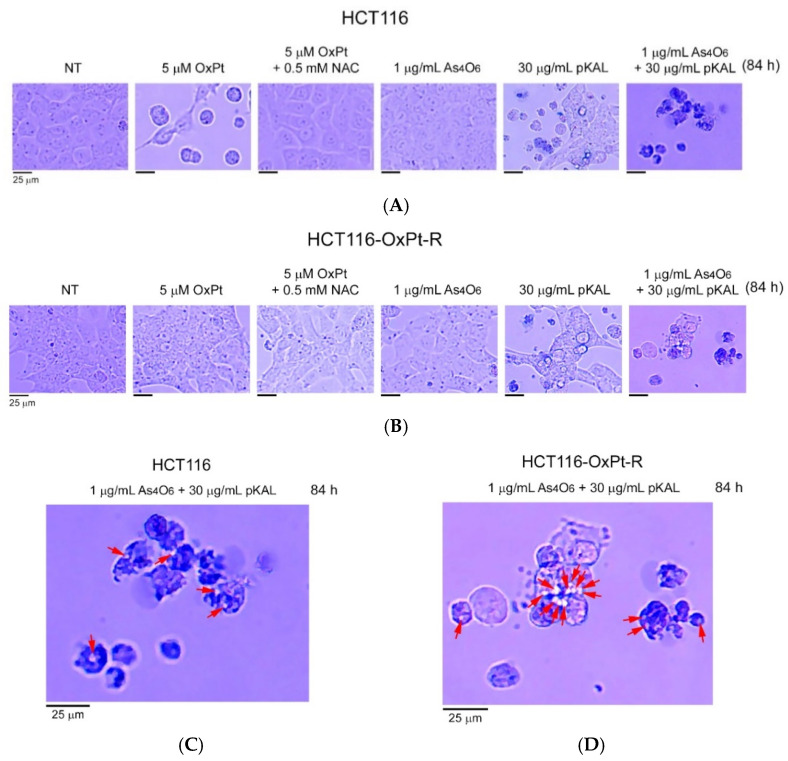
Dead cell morphology after co-treatment with As_4_O_6_ and pKAL in HCT116 and HCT116-OxPt-R cells: (**A**–**D**) Cells were cultured in a 6-well plate for 20 h, and then treated with the indicated concentrations of drugs for 84 h. Dead cell morphology was analyzed by phase-contrast microscopy after trypan blue staining. Representative intracellular vesicles are indicated by arrows (**C**,**D**).

To better understand the anticancer properties of combined treatment with As_4_O_6_ and pKAL in HCT116 and HCT116-OxPt-R cells, the morphology of dead cells was analyzed by phase-contrast microscopy after trypan blue staining. As a result, trypan blue-stained dead cells were detected in both HCT116 and HCT116-OxPt-R cells treated with 30 μg/mL pKAL for 84 h, and this effect was more prominent in the case of co-treatment with 1 μg/mL As_4_O_6_ and 30 μg/mL pKAL for 84 h (Figure 6A,B, panels 5, 6). Very interestingly, our results demonstrated that combined treatment with 1 μg/mL As_4_O_6_ and 30 μg/mL pKAL induced intracellular vesicles in the trypan blue-stained dead cells in both cancer cells, as indicated by arrows (Figure 6C,D). These results suggest that the synergistic anticancer effect of As_4_O_6_ and pKAL is associated with the increase in intracellular vesicles that may promote cell death processes in both HCT116 and HCT116-OxPt-R cells.

### 2.4. Effect of NAC on the Anticancer Effect of Co-Treatment with As_4_O_6_ and pKAL: Differences in the Mechanism of Combined Anticancer Efficacy of As_4_O_6_ and pKAL According to Treatment Period

**Figure 7:**
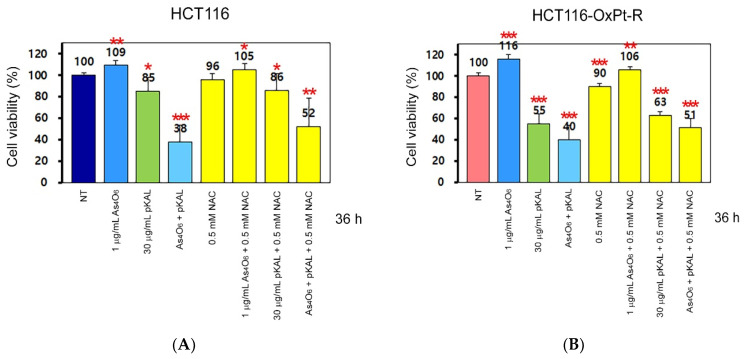
Effect of NAC on the regulation of cytotoxicity by As_4_O_6_ and pKAL in HCT116 and HCT116-OxPt-R cells: (**A**,**B**) Cells were grown on a 24-well plate for 20 h, treated with the indicated concentrations of drugs for 36 h, and analyzed by the CCK-8 assay. Statistical significance was determined using Student’s *t*-test: * *p* < 0.05; ** *p* < 0.005; *** *p* < 0.001.

In Figure 4, Figure 5 and Figure 6, our results show that the morphological changes induced by 5 μM OxPt were significantly inhibited in HCT116 cells in the presence of 0.5 mM NAC. These results prompted us to investigate how the cytotoxicity induced by combined treatment with As_4_O_6_ and pKAL is regulated by NAC using the CCK-8 assay. As shown in Figure 7A,B, the cell viability was slightly increased by treatment with 1 μg/mL As_4_O_6_ for 36 h in both HCT116 cells (109%) and HCT116-OxPt-R cells (116%). This suggests that survival or proliferation signaling may be partially activated during the treatment with 1 μg/mL As_4_O_6_ for 36 h. In addition, the cytotoxicity of 30 μg/mL pKAL was higher in HCT116-OxPt-R cells (55%) than in HCT116 cells (85%), and this effect was remarkably enhanced by co-treatment with As_4_O_6_ and pKAL in both HCT116 (38%) and HCT116-OxPt-R cells (40%). Notably, the cytotoxicity of 30 μg/mL pKAL (85%) alone in HCT116 cells was not significantly affected by 0.5 mM NAC (86%), whereas the cytotoxicity of 30 μg/mL pKAL (55%) alone in HCT116-OxPt-R cells was slightly inhibited by co-treatment with 0.5 mM NAC (63%). Moreover, the cytotoxicity induced by co-treatment with As_4_O_6_ and pKAL in HCT116 (38%) and HCT116-OxPt-R cells (40%) was somewhat significantly inhibited by 0.5 mM NAC in both HCT116 (52%) and HCT116-OxPt-R cells (51%). These results suggest that the cooperative anticancer effect of As_4_O_6_ and pKAL is partly related to ROS-mediated cytotoxicity.

To further elucidate the effect of NAC on the cooperative anticancer mechanism induced by co-treatment with pKAL and As_4_O_6_ in HCT116 and HCT116-OxPt-R cells, we analyzed the changes in proteins involved in autophagy, apoptosis, survival, death, and ROS signaling by Western blot and densitometry analysis. p62 (also known as Sequestosome 1 or SQSTM1) plays a crucial role in the regulation of apoptosis, autophagy, and inflammation, through its multiple functional domains; representatively, the LIR domain is required for binding to LC3, which is essential for autophagosome formation [26]. p62 interacts with LC3, a protein which is crucial for targeting the aggregates to the autophagosome [27,28]. In addition, p62 and LC3 are known to be upregulated in response to ROS and DNA damage [29].

**Figure 8:**
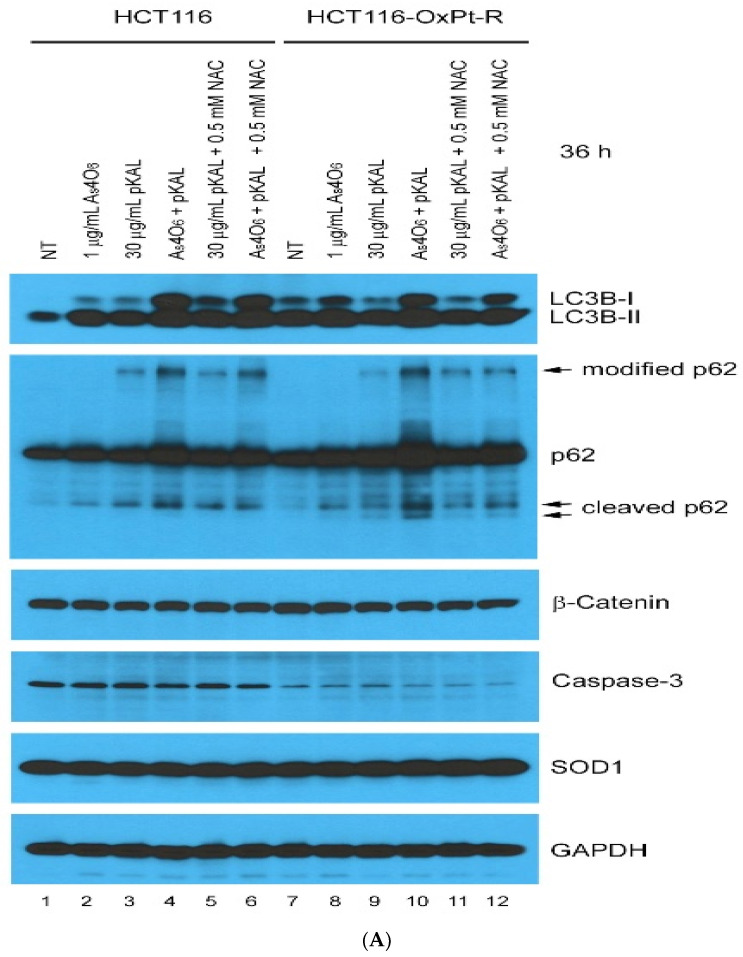

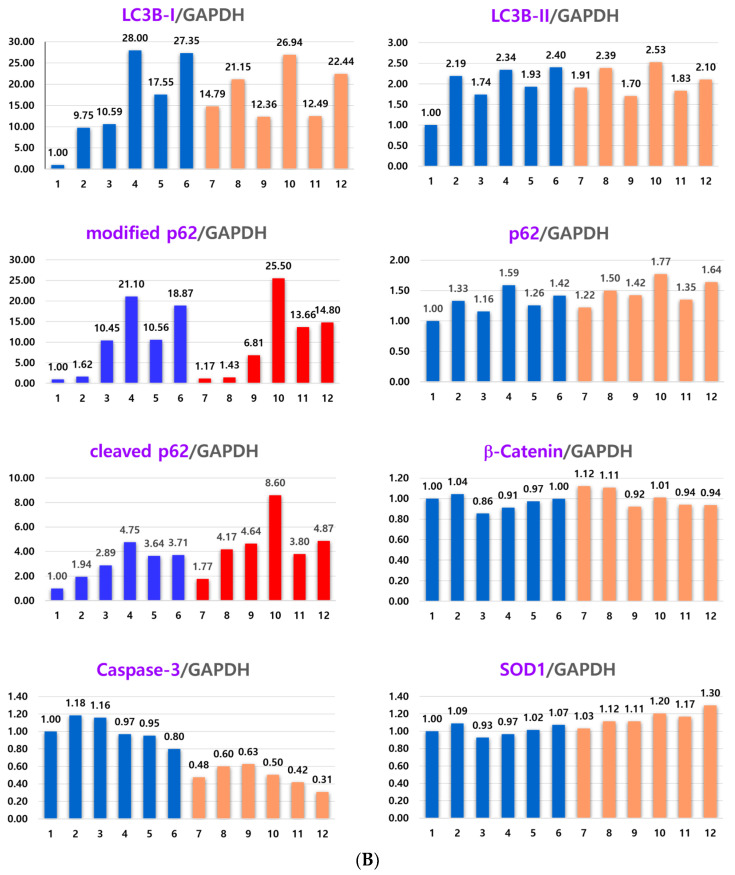
Effect of NAC on the anticancer mechanism of As_4_O_6_ and pKAL in HCT116 and HCT116-OxPt-R cells: (**A**) Cells were grown for 20 h on a 10 cm dish, and then treated with the indicated concentrations of drugs for 36 h. Whole-cell extracts were analyzed by Western blot using the indicated antibodies. (**B**) Densitometry analysis of protein bands in Figure 8A was conducted using the ImageJ program.

In Figure 8, our results show that LC3B-I and LC3B-II were significantly upregulated by co-treatment with As_4_O_6_ and pKAL for 36 h compared to treatment with 1 μg/mL As_4_O_6_ or 30 μg/mL pKAL alone in both cells (lanes 4, 10), suggesting that the cooperative anticancer effect of As_4_O_6_ and pKAL is related to enhanced autophagic activities. This phenomenon was not significantly inhibited by 0.5 mM NAC in both cancer cells. Moreover, after co-treatment with As_4_O_6_ and pKAL for 36 h, p62 and cleaved p62 were significantly upregulated in both cells compared to treatment with As_4_O_6_ or pKAL alone. However, the above results showed different responses to 0.5 mM NAC treatment: the increase in p62 by the combination treatment was not significantly reduced by 0.5 mM NAC treatment, but the increase in cleaved p62 by the combination treatment was significantly reduced by 0.5 mM NAC treatment. This finding suggests that the increase in p62 and cleaved p62 may have occurred by different mechanisms. To test the association with oxaliplatin resistance, we evaluated the effects on previously oxaliplatin-resistance-related proteins, β-catenin, superoxide dismutase [Cu-Zn] (SOD1), and caspase-3, but no particular effects were found on these protein expressions (caspase-3 and SOD1 panels). This is consistent with the finding that the combination therapy of As_4_O_6_ and pKAL showed similar results in HCT 116 and HCT116-OxPt-R cells.

**Figure 9:**
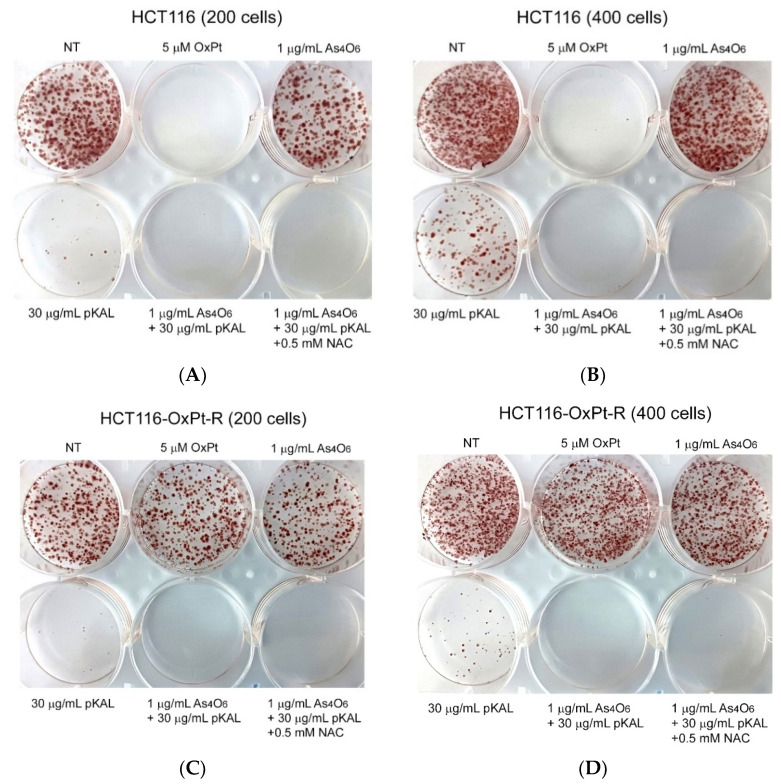
Long-term effects of OxPt, As_4_O_6_, pKAL, and NAC on the colony formation ability of HCT116 and HCT116-OxPt-R cells: (**A**–**D**) 200 and 400 cells were grown for 20 h on a 6-well plate, and then treated with the indicated concentrations of drugs for 60 h. After changing the medium, the cells were continuously grown for 9 days, and the produced colonies were detected by hematoxylin staining.

To further elucidate the effect of NAC on the long-term anticancer effect of pKAL and As_4_O_6_, a colony formation assay was performed in 200 or 400 HCT116 and HCT116-OxPt-R cells. As expected, the colony formation ability of HCT116 cells was almost blocked by the long-term anticancer effect of 5 μM OxPt, whereas no anticancer effect of 5 μM OxPt was observed in HCT116-OxPt-R cells (Figure 9A–D, NT and 5 μM OxPt wells). In addition, the colony formation ability of HCT116 and HCT116-OxPt-R cells was not significantly regulated by 1 μg/mL As_4_O_6_, but was markedly inhibited by 30 μg/mL pKAL (Figure 9A–D, NT, 1 μg/mL As_4_O_6_, and 30 μg/mL pKAL wells). Moreover, the colony formation ability of HCT116 and HCT116-OxPt-R cells was more markedly inhibited by co-treatment with 1 μg/mL As_4_O_6_ and 30 μg/mL pKAL than treatment with 1 μg/mL As_4_O_6_ or 30 μg/mL pKAL alone (Figure 9A–D). However, the cooperative anticancer effect of As_4_O_6_ and pKAL in blocking the colony formation ability of HCT116 and HCT116-OxPt-R cells was not significantly affected by 0.5 mM NAC (Figure 9A–D). These results suggest that the cooperative anticancer effect of long-term co-treatment with As_4_O_6_ and pKAL can inhibit the colony formation ability of HCT116 and HCT116-OxPt-R cells in an ROS-independent manner, and this shows that the ROS dependency disappears as the treatment time increases (long-term treatment).

**Figure 10:**
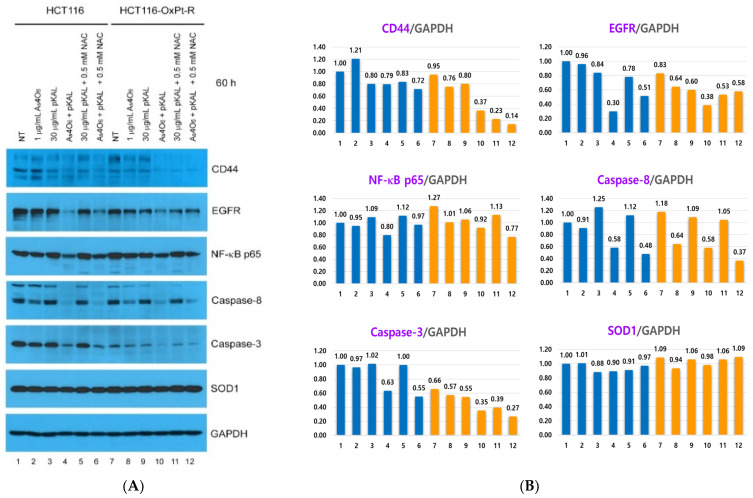

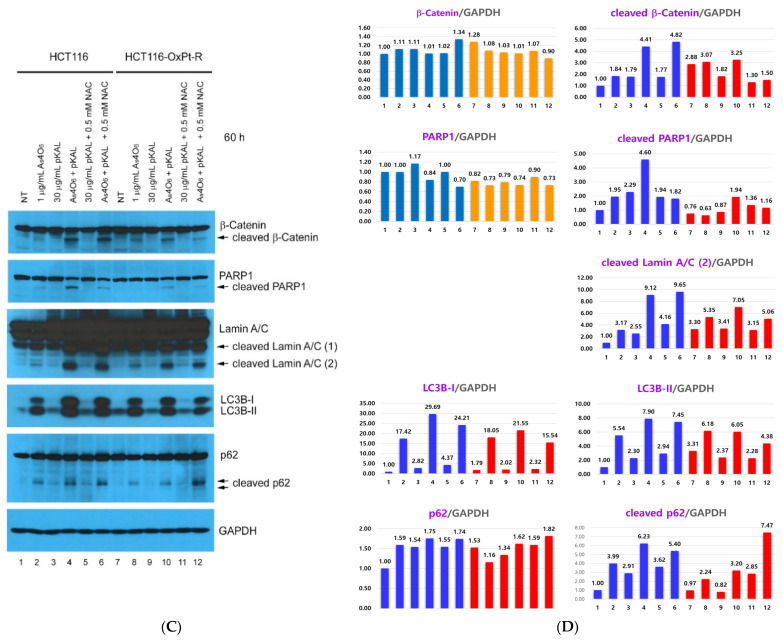
Effect of NAC on the anticancer mechanism of As_4_O_6_ and pKAL in HCT116 and HCT116-OxPt-R cells: (**A**,**C**) Cells were grown for 20 h on a 10 cm dish, and then treated with the indicated concentrations of drugs for 60 h. Whole-cell extracts were analyzed by Western blot using the indicated antibodies. (**B**,**D**) Densitometry analysis of Figure 10A,C).

Next, to confirm that the anticancer efficacy of the combination treatment of pKAL and As_4_O_6_ varied depending on the treatment time and ROS dependence, and to further confirm the therapeutic mechanism of the anticancer efficacy of the combination treatment, Western blot and densitometry analyses were performed on protein candidates related to the increased anticancer efficacy of the co-treatment with pKAL and As_4_O_6_ in Figure 3. As shown in Figure 10A,B, the stem cell marker CD44 antigen (CD44) was significantly downregulated by co-treatment with As_4_O_6_ and pKAL for 60 h in HCT116-OxPt-R cells compared to by 1 μg/mL As_4_O_6_ or 30 μg/mL pKAL alone, but this synergistic effect was not inhibited by 0.5 mM NAC. The cell survival proteins EGFR and NF-κB p65 were significantly downregulated by co-treatment with As_4_O_6_ and pKAL for 60 h in both cells, and this phenomenon was inhibited by 0.5 mM NAC. This suggests that the cooperative anticancer effect of As_4_O_6_ and pKAL may be exerted through the downregulation of EGFR and NF-κB p65 in an ROS-dependent manner. In addition, the proapoptotic proteins caspase-8 and caspase-3 were significantly downregulated by combined treatment with As_4_O_6_ and pKAL for 60 h in both cells, but this phenomenon was not inhibited by 0.5 mM NAC. This suggests that the cooperative anticancer effect of As_4_O_6_ and pKAL in HCT116 cells may be associated with the activation of caspases in an ROS-independent manner. Furthermore, the results in Figure 9C,D show that the cleavage of β-catenin, PARP1, lamin A/C, and p62 was markedly induced by combined treatment with As_4_O_6_ and pKAL for 60 h in both cells, and these phenomena, except for cleaved p62, were inhibited by 0.5 mM NAC. Unlike p62, the upregulation of LC3B-I and LC3B-II by combined treatment with As_4_O_6_ and pKAL for 60 h was somewhat inhibited by 0.5 mM NAC in both cells. As shown in Figure 10, the therapeutic effect of 0.5 mM NAC on p62 and upregulation of LC3B-I and LC3B-II after co-treatment with As_4_O_6_ and pKAL for 60 h was not significant, while the cleaved form of p62 was reduced by 0.5 mM NAC in the 36 h co-treatment.

Taken together, our results suggest that the cooperative anticancer effect of As_4_O_6_ and pKAL may be associated with ROS-dependent and ROS-independent regulation of various cell survival and death determinants. Although the synergistic anticancer effect of As_4_O_6_ and pKAL was shown to occur in an ROS-dependent or ROS-independent manner, these changes were accompanied by upregulation of p62 and LC3B-I/LC3B-II, suggesting that they may be related to autophagic cell death. This is also thought to be related to the increase in intracytoplasmic vesicles and nuclear deformation induced by co-treatment with As_4_O_6_ and pKAL compared to the treatment of each drug alone. In addition, co-treatment with As_4_O_6_ and pKAL activated caspases and induced cleavages of PARP and lamin A/C.

### 2.5. Confirmation of Autophagic Cell Death Relevance: Effect of Wortmannin (Wort) on the Cooperative Anticancer Effect of As_4_O_6_ and pKAL

To confirm the involvement of the cooperative anticancer mechanism of As_4_O_6_ and pKAL in autophagic cell death, cytotoxicity of co-treatment with As_4_O_6_ and pKAL was evaluated with the CCK-8 assay after treatment with Wort. Wort, an inhibitor of PI3-kinases, affects DNA damage signaling by interfering with γ-H2AX, a marker of DNA double-strand breaks (DSBs) [30,31], and it can effectively block autophagy in vitro and in vivo [31,32,33]. Specifically, Wort inhibits autophagy by interfering with autophagosome formation, which is essential for the autophagy process [32].

**Figure 11:**
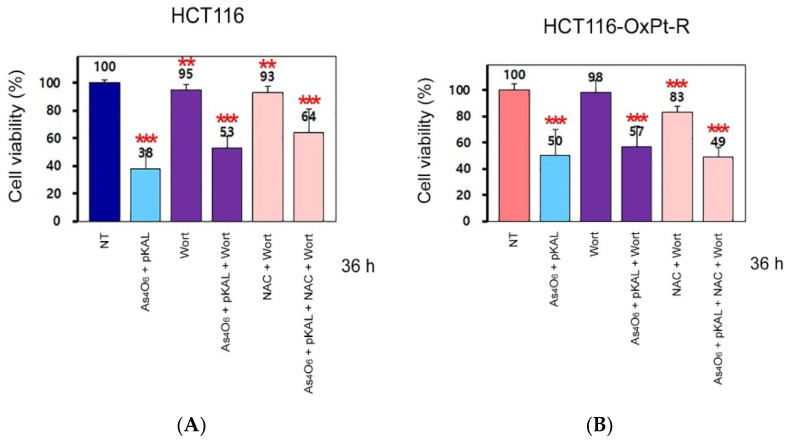
Effect of Wort on the cooperative cytotoxicity of As_4_O_6_ and pKAL in HCT116 and HCT116-OxPt-R cells: (**A**,**B**) Cells were grown for 20 h on a 24-well plate, treated with the indicated drugs (1 μg/mL As_4_O_6_; 30 μg/mL pKAL; 0.5 μM Wort; 0.5 mM NAC) for 36 h, and then analyzed by the CCK-8 assay. Statistical significance was determined using Student’s *t*-test: ** *p* < 0.005; *** *p* < 0.001.

Here, we tested the effects of Wort on the anticancer effects of combined treatment with As_4_O_6_ and pKAL. As shown in Figure 11A,B, combined treatment with As_4_O_6_ and pKAL for 36 h significantly reduced cell viability in both HCT116 (38%) and HCT116-OxPt-R cells (50%), and this phenomenon was inhibited by 0.5 μM Wort in both HCT116 (53%) and HCT116-OxPt-R cells (57%). This finding suggests that the cooperative anticancer effect of As_4_O_6_ and pKAL may be related to autophagic cell death.

**Figure 12:**
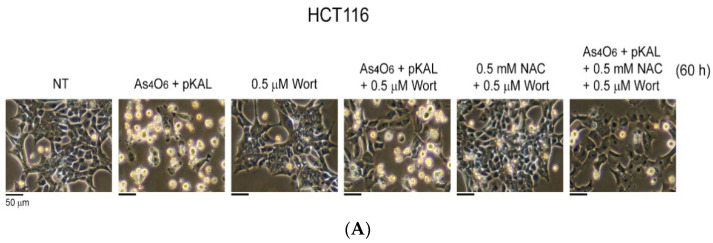

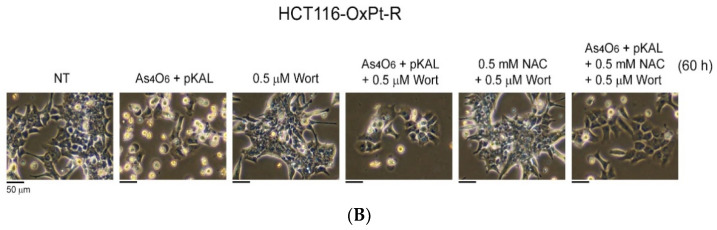
Effect of Wort and NAC on the cooperative morphological changes induced by As_4_O_6_ and pKAL in HCT116 and HCT116-OxPt-R cells: (**A**,**B**) Cells were grown for 20 h on a 10 cm dish, treated with the indicated drugs (1 μg/mL As_4_O_6_; 30 μg/mL pKAL; 0.5 μM Wort; 0.5 mM NAC) for 60 h, and then analyzed by phase-contrast microscopy.

Next, the effect of Wort on the cell morphological changes induced by the cooperative anticancer effect of co-treatment with As_4_O_6_ and pKAL was evaluated using phase-contrast microscopy. As shown in Figure 12A,B, the co-treatment of As4O6 and pKAL for 60 h in both HCT116 and HCT116-OxPt-R cells significantly induced morphological changes in dead cells (panels 1 and 2), and this phenomenon was partially but significantly inhibited in the presence of 0.5 µM Wort, especially in HCT116 cells (panels 2 and 4). Interestingly, the cooperative anticancer effects of As_4_O_6_ and pKAL were more significantly inhibited by co-treatment with NAC and Wort than by Wort alone in both HCT116 and HCT116-OxPt-R cells (panels 4 and 6). In addition, the formation of intracytoplasmic vesicles was significantly reduced in the presence of 0.5 µM Wort +/− NAC in both HCT116 and HCT116-OxPt-R cells. These results suggest that the cooperative anticancer effect of As_4_O_6_ and pKAL is related to autophagic cell death and that other cell deaths related to ROS generation may exist.

### 2.6. Confirmation of Autophagic Cell Death Relevance and Other Cell Death Possibilities: Effect of Wortmannin (Wort) and NAC on the Cooperative Anticancer Effect of As_4_O_6_ and pKAL

**Figure 13:**
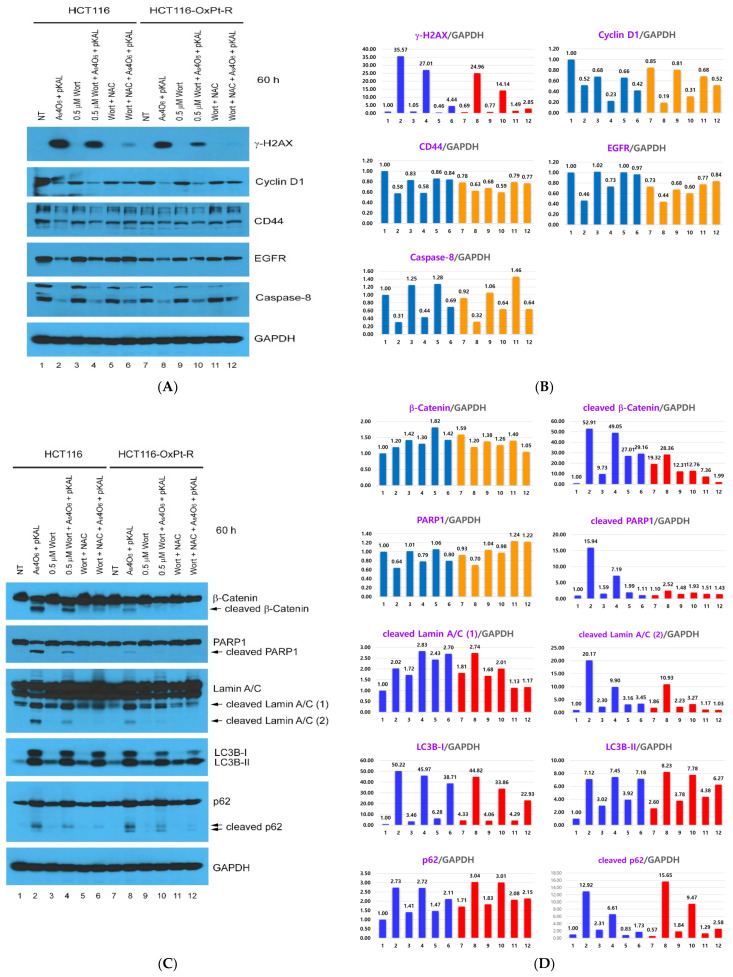
Effect of Wort + NAC on the cooperative anticancer mechanism of As_4_O_6_ and pKAL in HCT116 and HCT116-OxPt-R cells: (**A**,**C**) Cells were grown for 20 h on a 10 cm dish, and then treated with the indicated drugs (1 μg/mL As_4_O_6_; 30 μg/mL pKAL; 0.5 μM Wort; 0.5 mM NAC) for 60 h. Whole-cell extracts were analyzed by Western blot using the indicated antibodies. (**B**,**D**) Densitometry analysis of Figure 13A,C.

In order to better understand the effect of Wort and/or Wort + NAC on the cooperative anticancer mechanism of pKAL and As_4_O_6_ in HCT116 and HCT116-OxPt-R cells, we examined the change in protein expressions related to DNA damage, cell cycle, survival, and death signaling by Western blot and densitometry analysis. As shown in Figure 13A,B, the DNA damage marker γ-H2AX, a phosphorylated form of histone H2AX on serine 139, was more significantly upregulated by co-treatment with As_4_O_6_ and pKAL for 60 h in HCT116 cells than in HCT116-OxPt-R cells (lanes 1, 2 and 7, 8), and this phenomenon was inhibited by 0.5 μM Wort in both cells (lanes 2, 4 and 8, 10), and further inhibited by 0.5 μM Wort + 0.5 mM NAC (lanes 4, 6 and 10, 12). In contrast, cell cycle regulator cyclin D1, survival regulators CD44 and EGFR, and cell death regulator caspase-8 were significantly downregulated by co-treatment with As_4_O_6_ and pKAL for 60 h in both cells (lanes 1, 2 and 7, 8), and this phenomenon was inhibited by Wort in both cells (lanes 2, 4 and 8, 10), and further inhibited by Wort + NAC (lanes 4, 6 and 10, 12). Similarly to γ-H2AX, the results of Figure 13C,D show that autophagosome markers LC3B-I, LC3B-II, and p62 were significantly upregulated by co-treatment with As_4_O_6_ and pKAL for 60 h in both cells (lanes 1, 2 and 7, 8), and this phenomenon was inhibited by Wort in both cells (lanes 2, 4 and 8, 10), and further inhibited by Wort + NAC (lanes 4, 6 and 10, 12). In addition, the cleavage of β-catenin, PARP1, and lamin A/C was more significantly increased by co-treatment with As_4_O_6_ and pKAL for 60 h in HCT116 cells than in HCT116-OxPt-R cells, whereas the cleavage of p62 was more significantly increased by co-treatment with As_4_O_6_ and pKAL in HCT116-OxPt-R cells than in HCT116 cells. Moreover, the cleavage of β-catenin, PAPR1, lamin A/C, and p62 induced by combined treatment of As_4_O_6_ and pKAL was inhibited by Wort in both cells (lanes 2, 4 and 8, 10), and further inhibited by Wort + NAC (lanes 4, 6 and 10, 12). These results demonstrate that the combined treatment with As_4_O_6_ and pKAL in HCT116-OxPt-R cells can activate more potent cell death signaling by inducing not only the cleavage of β-catenin, PAPR1, and lamin A/C but also the cleavage of p62, and the cooperative anticancer effect of As_4_O_6_ and pKAL can occur by modulating proteins playing important roles in DNA damage, the cell cycle, survival, death, and autophagy associated with PI3K and ROS signaling in both HCT116 and HCT116-OxPt-R cells. Taken together, these results also support that the cooperative anticancer effect of As_4_O_6_ and pKAL is related to autophagic cell death and that other cell deaths related to ROS generation may exist.

## 3. Discussion

This study was designed to confirm the synergistic effect between As_4_O_6_ and pKAL, and then investigate the mechanisms for the anticancer effects of As_4_O_6_ and pKAL combination treatment. We found that As_4_O_6_ enhanced the anticancer effects of pKAL, which showed anticancer effects in drug-resistant and radiation-resistant cells. Here, we clearly demonstrated that ROS and autophagic activity were important in generating anticancer effects induced by co-treatment with As_4_O_6_ and pKAL as well as downregulation of EGFR, CD44, and cyclin D1 by showing that the cooperative anticancer effects of As_4_O_6_ and pKAL were significantly associated with the increase in intracellular vesicles and the cleaved form of p62 through the regulation of key proteins involved in the induction of DNA damage, inhibition of the cell cycle, suppression of cell survival signaling, and promotion of cell death signaling in both HCT116 and HCT116-OxPt-R colorectal cancer cells.

Initially, it was inferred that the synergistic effect between As_4_O_6_ and pKAL may be related to the OxPt-resistance-related protein because pKAL had a greater anticancer effect in HCT116-OxPt-R cells than in parental HCT116 cells, and the co-treatment of As4O6 and PKAL showed a synergistic anticancer effect in HCT116-OxPt-R cells (Figure 2, Figure 7 and Figure 11). However, as in this study, there was no significant difference in the increase in the anticancer efficacy of As_4_O_6_ between HCT 116 cells and HCT116-OxPt-R cells, although pKAL showed a greater anticancer effect in HCT116-OxPt-R cells than in parental HCT116 cells, and As_4_O_6_ increased the anticancer efficacy of pKAL in both HCT116 and HCT116-OxPt-R cells. As_4_O_6_ and PKAL combination treatment increased the anticancer efficacy by regulating genes related to cell survival and resistance such as EGFR, NF-kB, and CD44 rather than SOD1 and β-catenin, which are related to OxPt-R-resistance-related proteins [8].

Regarding NF-kB, it is a very important transcription factor that is related to cancer cell growth, invasion, metastasis, and drug resistance [34]. However, pKAL did not suppress NF-kB activities in both HCT116 and HCT116-OxPt-R cells [25]. In addition, growth factors and cytokines involved in cell survival signaling were upregulated under pKAL-induced cell death circumstances; specifically, the upregulation of NF-κB due to pKAL was involved in the inhibition of pKAL-induced cell death [14]. However, co-treatment with As_4_O_6_ and PKAL significantly inhibited the expression of NF-kB p65 in both HCT116 and HCT116-OxPt-R cells. It is thought that the suppression of upregulated NF-kB by As_4_O_6_ is helpful in enhancing the anticancer effect. In particular, we previously reported that As_4_O_6_ exhibited an anticancer effect by inhibiting NF-kB p65 activity [24]. In addition, cyclin D1 and AKT are highly associated with NF-κB activity [34,35]. This finding supports that one of the mechanisms for the anticancer effects induced by co-treatment with As_4_O_6_ and pKAL is suppression of NF-kB associated with cell survival signaling activities [24].

Next, EGFR is also a famous receptor involved in cancer cell survival, proliferation, and migration and metastasis [36]. Even though EGFR was not a candidate OxPt-R-related protein in a previous study [25], EGFR expression is significantly suppressed by co-treatment with As_4_O_6_ and pKAL. In the previous study, pKAL suppressed EGFR in a dose-dependent manner [25]. As_4_O_6_ contributes to amplifying the effect of pKAL in reducing EGFR expression. In addition, in the previous study [25] and this study (Figure 10 and Figure 13), since the expression of EGFR at baseline was higher in HCT116 cells than in HCT116-OxPt-R cells, the magnitude of the effect of As_4_O_6_ and pKAL combination therapy in reducing EGFR expression was greater in HCT116 cells. This may be the reason why the anticancer effect of pKAL in HCT116 cells, although small, increases more significantly in HCT116-OxPt-R cells during combination therapy with As_4_O_6_ and pKAL (Figure 2, Figure 7 and Figure 11). This finding supports that EGFR reduction is another mechanism for the increased anticancer efficacy of As_4_O_6_ and pKAL combination therapy.

Cyclin D1 is a positive regulator of the cell cycle and promotes G1-to-S-phase transition in cooperation with CDK4 or 6. Amplification of the gene and overexpression of cyclin D1 protein have frequently been found in several types of human malignant neoplasms. Cyclin D1 is related to cancer aggressiveness [37]. It is a direct target of mitogen-activated protein kinase cascades including EGFR [38], and phosphatidylinositol 3-kinase (PI3K)-Akt [39], and it is also regulated by transcription factors such as NF-κB [35]. The expression pattern of cyclin D1 is the same as that of EGFR (Figure 10 and Figure 13), and this is also thought to be another mechanism for the increased anticancer efficacy of As_4_O_6_ and pKAL combination therapy.

CD44 has been implicated as a cancer stem cell marker in several malignancies of hematopoietic and epithelial origin. It is involved in cancer cell proliferation, adhesion, migration, invasion, and even drug resistance. The expression pattern of CD44 is the same as that of EGFR (Figure 10 and Figure 13), and this is also thought to be another mechanism for the increased anticancer efficacy of As_4_O_6_ and pKAL combination therapy.

Since β-catenin was a candidate protein for OxPt-R in a previous study [25], we observed it closely and experimented on it several times, but the pattern of the decrease in β-catenin expression was different from the expression of EGFR, cyclin D1, and CD44 mentioned above, and the decrease in protein amount was mainly judged to be protein cleavage rather than a decrease in expression (Figure 10 and Figure 13). In addition, the pattern of the decrease in β-catenin expression is similar to the expression pattern of the cleavage of PARP1, lamin A/C, and p62 (Figure 10 and Figure 13), so it is thought to be a by-product of the progression of cell death.

Regarding ROS and DNA damage response, moderately high levels of ROS are beneficial to maintaining tumorigenesis, while toxic levels of ROS could be an important force in killing cancer cells. ROS have become an important anticancer target based on the proapoptotic effect of toxic levels of ROS [40]. As the cell death and DNA damage response (increase in DNA damage marker γ-H2AX) induced by As_4_O_6_ and pKAL co-treatment are reduced by NAC treatment (Figure 7, Figure 11 and Figure 13), it seems that cell death related to the generation of ROS is one mechanism of As_4_O_6_ and pKAL combination therapy. In particular, it is well known that arsenic compounds as well as As_4_O_6_ induce cell death related to ROS generation [41], and the fact that cell death induced by As_4_O_6_, pKAL, and Wort co-treatment is reduced by NAC treatment also supports that one of the mechanisms for the anticancer effects induced by co-treatment with As_4_O_6_ and pKAL is ROS generation and DNA damage. However, the anticancer activity reduction effect induced by the co-treatment of As4O6 and pKAL with NAC decreased with increasing treatment time, and the cell death effect induced by the co-treatment of As4O6 and pKAL was also reduced by the treatment of Wort (Figure 7, Figure 11 and Figure 13). Considering this, the anticancer effect induced by co-treatment with As_4_O_6_ and pKAL implies that there is an anticancer mechanism independent of ROS generation.

However, in this study, we did not investigate further why the anticancer effect of As_4_O_6_ and pKAL became independent of ROS generation over time. In addition, the fact that the actual ROS production was not directly shown to be increased by co-treatment with As_4_O_6_ and pKAL, but instead explained the ROS relationship under NAC treatment, suggests that additional experiments should be added to elucidate the relationship between the expression changes in NF-kB, cyclin D1, AKT, and EGFR and ROS generation induced by the combination treatment. The reason why further experiments are required is that N-acetylcysteine (NAC) is often used as an antioxidant to suppress ROS and prevent cell death, but it can also induce excessive ROS production and oxidative stress, which can induce cell death, especially in certain cancer cells [42]. These effects depend on the concentration of NAC and the specific cell type. The effect of NAC can be concentration-dependent. High concentrations (higher than 5 mM) may reduce cell death by scavenging ROS, while lower concentrations (lower than 1 mM) may paradoxically enhance cell death by increasing ROS levels [42]. Based on the above results, it seems that high concentrations of NAC should be used, but the reason why low concentrations of NAC were selected in this study is that this study required long-term treatment (36–84 h). This is because the longer the administration time is, the more it has the same effect as a high concentration [43], and when treatment with a high concentration of NAC occurs for more than 12 h, it causes changes in the expression of various genes and cell morphology [44].

Regarding intracellular cytoplasmic vesicles induced by co-treatment with As_4_O_6_ and pKAL, we investigated the possibility of autophagic cell death. Cleavage of PARP1, p62, and other proteins (β-catenin and lamin A/C) was shown as evidence of cell death [45,46], which was accompanied by the progression of autophagy (upregulation of LC3B and p62). In addition, the decrease in cell death and intracellular cytoplasmic vesicles with Wort, an inhibitor of autophagosome formation, was additional evidence that the anticancer effect of co-treatment with As4O6 and pKAL is involved in autophagic cell death.

Lastly, regarding caspase-induced apoptosis, PARP1 is cleaved by caspases, primarily caspase-3 and caspase-7, at the DEVD214 site, resulting in the formation of 24 kDa and 89 kDa fragments [47]. This cleavage inactivates PARP1’s DNA repair function, potentially contributing to cell death processes. Therefore, co-treatment with As4O6 and pKAL is involved in caspase-induced apoptosis. However, whether the caspase-induced apoptosis induced by co-treatment with As_4_O_6_ and pKAL is related to autophagic cell death is unclear, because in autophagy-induced cell death, PARP1 is cleaved by caspases, and the interaction between autophagy and apoptosis is complex [48].

A limitation is that we lack direct evidence of ROS generation by co-treatment with As_4_O_6_ and pKAL as mentioned above and verification of how the proposed anticancer efficacy mechanisms of the co-treatment with As_4_O_6_ and pKAL are intertwined, so further studies are needed. In addition, to determine the exact identity of intracellular vesicles, it is recommended to perform an immunofluorescence assay using LC3 and p62. However, this study indirectly replaced this assay with Western blot analysis for LC3 and p62, which can be considered another limitation.

A limitation of this study is that many proteins are involved in the anticancer effects of the combined treatment of As_4_O_6_ and pKAL, and further studies are needed to elucidate the relationship between the proteins that determine the anticancer efficacy in parental HCT116 and HCT116-OxPt-R cells. In addition, to provide evidence for ROS generation by the combination treatment, NAC was used instead of direct ROS measurement (e.g., using DCFH-DA). In addition, in order to accurately confirm whether intracellular vesicles are autophagosomal, immunofluorescence analysis using LC3 and p62 would be better than Western blot analysis with LC3 and p62. However, this study was conducted to find a way to overcome the phenomenon of decreasing anticancer efficacy of pKAL over time in parental HCT116 cells [25], and as shown in this study, As4O6 not only solved this problem by increasing the anticancer efficacy of pKAL, but also increased the anticancer efficacy of the combination treatment in HCT116-OxPt-R cells.

## 4. Materials and Methods

### 4.1. Materials

RPMI-1640 medium (+ 25 mM Hepes, + L-Glutamine) was obtained from HyClone (Logan, UT, USA). Penicillin–Streptomycin (10,000 U/mL), TrypLE^TM^ Express Enzyme with phenol red, and HPLC-grade water were obtained from Thermo Fisher Scientific (Grand Island, NY, USA). N-Acetyl-L-cysteine (NAC), wortmannin, Brilliant Blue G, and PBS (pH 7.4) were obtained from Sigma-Aldrich (St. Louis, MO, USA). Cell counting kit-8 (CCK-8) and D-Plus^TM^ CCK cell viability assay reagents were obtained from Dojindo (Kumamoto, Japan) and Dongin LS (Daejeon, Republic of Korea), respectively. In addition, 30% acrylamide/bis solution 29:1 was obtained from Bio-Rad (Hercules, CA, USA). Tween-20 was obtained from Amresco (Solon, OH, USA), and 0.22 μm nitrocellulose (NC) transfer membrane was obtained from GVS Life Sciences (Sanford, ME, USA). The D-Plus^TM^ ECL pico solution kit was obtained from Dongin LS (Daejeon, Republic of Korea). X-ray film (CP-BU NEW) was obtained from AGFA (Mortsel, Belgium), and 4% formaldehyde solution was obtained from Merck KGaA (Darmstadt, Germany). Hematoxylin solution was obtained from TissuePro Technology (Gainesville, FL, USA), and 0.4% trypan blue solution was obtained from Bioworld (Louis Park, MN, USA). Dishes, plates, tubes, pipettes, and the 96-well ImmunoPlate for cell culture were obtained from SPL Life Sciences (Pocheon, Republic of Korea) or Thermo Fisher Scientific (Rockford, IL, USA). p53 (DO-1), β-catenin (E-5), ERK1 (K-23), caspase-8 (8CSP03), PARP1 (F-2), cyclin-D1 (A-12), EGFR (1005), Akt1/2/3 (H-136), NF-κB p65 (F-6), caspase-3 (E-8), SOD1 (FL-154), lamin A/C (E-1), and GAPDH (FL-335) antibodies were obtained from Santa Cruz Biotechnology (Santa Cruz, CA, USA). LC3B (ab51520) and CD44 (EPR1013Y) antibodies were obtained from Abcam Biotechnology (Cambridge, UK). p62 lck ligand (Sequestosome-1) antibody was obtained from BD Biosciences (San Jose, CA, USA). Phospho-Ser139-H2AX (γ-H2AX) antibody was obtained from Upstate Biotechnology (Lake Placid, NY, USA). Secondary goat anti-rabbit and anti-mouse HRP conjugates were obtained from Bio-Rad (Hercules, CA, USA).

### 4.2. As_4_O_6_ and pKAL Compounds

As_4_O_6_ was obtained from Chonjisan institute (Seoul, Republic of Korea). The stock solution concentration was 1.15 g/mL. For experiments, As_4_O_6_ was dissolved in HPLC-grade water at a concentration of 20 mg/mL and stored at room temperature (RT) until use.

pKAL compounds were isolated from mixed tissues including roots, stems, leaves and flowers of *Artemisa annua* L. grown at Gaddongsook farm in Jinju, as previously reported [9,12,13]. To isolate pKAL compounds, the mixed tissues were lyophilized, ground, and extracted with 70% methanol at 60 °C for 20 h. The extract was filtered through a glass funnel and concentrated at 35 °C using a rotary evaporator. To remove fat components, the concentrated aqueous extract was extracted three times with equal volumes of *n*-hexane and methylene chloride. The filtrate was extracted three times with ethyl acetate to isolate the pKAL compounds and dried over anhydrous magnesium sulfate. pKAL compounds identified by liquid chromatography–tandem mass spectrometry (LC/MS/MS) are as follows: Caffeic acid, quercetin-3-O-galactoside, Mearnsetin-glucoside, kaempferol-3-O-glucoside, Ferulic acid, isorhamnetin-glucoside, Diosmetin-7-O-glucoside, Luteolin-7-O-glucoside, quercetin, Quercetagetin-3-O-methyl ether, luteolin, 8-methoxy-kaempferol, Quercetagetin-5,3-di-O-methyl ether, kaempferol, 3,5-dihydroxy-6,7,4′-trimethoxyflavone, 3,5-dihydroxy-6,7,3′,4′-tetramethoxyflavone, and isorhamnetin. The HPLC chromatogram of pKAL compounds was presented in a previous report [9]. For experiments, pKAL compounds were dissolved in DMSO solvent at a concentration of 100 mg/mL and stored in a −20 °C freezer until use.

### 4.3. Cell Culture

The HCT116 human colorectal cancer cell line was purchased from Korean Cell Line Bank (KCLB No. 10247). HCT116 cells were grown on a culture dish using maintenance medium A, which is RPMI medium containing L-glutamine (300 mg/L), 25 mM HEPES, 25 mM NaHCO_3_, 1% penicillin/streptomycin, and 10% heat-inactivated FBS (Thermo Fisher Scientific, Grand Island, NY, USA). Also, 5 μM of OxPt-resistant HCT116-OxPt-R cells was generated previously and grown on a culture dish using maintenance medium A with 5 μM OxPt [25]. Passages 21 to 34 of HCT116-OxPt-R cells were used in this study. HCT116 and HCT-OxPt-R cells were cultured at 37 °C in a humidified incubator supplemented with 5% CO_2_.

### 4.4. Phase-Contrast Microscopy

Cell morphology was analyzed by phase-contrast microscopy (EVOS XL Core, Life Technologies, Carlsbad, CA, USA) in a 10× objective (Inf Plan Achro 10× LWD PH, 0.25 NA/6.9 WD) with 150× amplification.

### 4.5. Western Blot Analysis

First, 1.5 × 10^6^ cells/10 mL medium were grown for 20 h on a 10 cm culture dish and then treated with drugs for the indicated times. After removing culture medium thoroughly, whole cells were extracted with 500 μL of 1× SDS sample buffer by spreading it well all over the dish. The whole-cell extract was boiled for 5 min at 95 °C and vortexed well, and this procedure was repeated once or twice until the extract become homogeneous. Proteins (2 μL each) were reacted for 30 min with 1 mL of Bradford reagent (0.05 g of Brilliant Blue G/24 mL of ethanol/50 mL of 85% phosphoric acid/426 mL of HPLC-grade water, which were resolved and filtered). The reaction solution (300 μL each) was transferred to a 96-well ImmunoPlate, and analyzed by measuring the absorbance at OD_595 nm_ using a microplate reader (SoftMax Pro 7). The resultant proteins (10 μg each in 15-well comb) were separated using 10 or 15% SDS-PAGE and transferred to an NC membrane at 20 mA for 16–18 h. After washing with PBST (0.1% Tween-20, PBS) twice for 1 h, the membrane was blocked for 30 min at RT in blocking buffer (3% skim milk/0.1% Tween-20/PBS) and then incubated with primary antibody in blocking buffer at 4 °C overnight. The blot was then washed with PBST twice for 15 min and incubated with an HRP-conjugated secondary antibody in blocking buffer for 2 h at RT. After being washed with PBST, the blot was analyzed by detection on an X-ray film using the D-Plus^TM^ ECL pico solution kit. For densitometry analysis, the film was scanned at high resolution into TIFF file format. The image was converted into JPEG file format using Photoshop CC 2018, the image mode was changed to grayscale, and protein bands were quantified using the ImageJ program bundled with 64-bit Java 8 over the internet (http://imagej.net/ij/download.html) (accessed on 16 February 2025).

### 4.6. Cell Viability Analysis

First, 4 × 10^4^ cells/1 mL medium were grown for 20 h on a 24-well plate and then treated with drugs for 36 h. After removing culture medium, cells were incubated with 150 μL of maintenance medium containing 10% CCK-8 (Dojindo) or D-Plus^TM^ CCK (Dongin LS) reagent for 1.5 h in a 37 °C CO_2_ incubator. The reaction solution (100 μL each) was then transferred to a 96-well ImmunoPlate and was analyzed by measuring the absorbance at OD_450 nm_ using a microplate reader (SoftMax Pro 7).

### 4.7. Phase-Contrast Microscopy of Hematoxylin-Stained Cells

First, 2 × 10^5^ cells/2 mL medium were grown for 20 h on a 6-well plate and then treated with drugs for the indicated times. Cells were washed with PBS, fixed with 4% formaldehyde solution at 4 °C overnight, washed with PBS twice, and then stained with 1 mL of hematoxylin solution for 7 h at RT by gentle shaking. After washing with PBS, 1 mL of 90% glycerol/PBS solution was added to each well, and then immediately analyzed by phase-contrast microscopy (EVOS XL Core, Life Technologies) in a 20× objective (Inf Plan Fluor 20× LWD, 0.45 NA/7.1 WD) with 300× amplification.

### 4.8. Phase-Contrast Microscopy of Trypan Blue-Stained Cells

First, 2 × 10^5^ cells/2 mL medium were grown for 20 h on a 6-well plate and then treated with drugs for 84 h. Without removing culture medium, 1 mL of 0.04% trypan blue solution was added to each well and incubated for 1 h at RT by gentle shaking. The cells were analyzed by phase-contrast light microscopy in a 20× objective (Inf Plan Fluor 20× LWD, 0.45NA/7.1WD) (EVOS XL Core, Life Technologies) with 300× amplification.

### 4.9. Colony Formation Assay

First, 200 or 400 cells/2 mL medium were grown for 20 h on a 6-well plate and then treated with drugs for 60 h. After changing to 4 mL of fresh medium, the cells were continuously grown for 9 days. The cells were washed with PBS, fixed with 2 mL of 4% formaldehyde solution at 4 °C overnight, and then washed with PBS twice and stained with 1 mL of hematoxylin solution at RT overnight by gentle shaking. After removing hematoxylin solution thoroughly, the 6-well plate was visualized by capturing it using a camera.

### 4.10. Statistical Analysis

Results for cell viability are presented as the mean ± standard deviation of the mean. Statistical significance between the control and sample was determined using Student’s *t*-test. Values of *p* < 0.05 are considered statistically significant.

## 5. Conclusions

To find a way to overcome the phenomenon of decreasing anticancer efficacy of pKAL over time in parental HCT116 cells [25], this study was designed to enhance the anti-cancer effects of pKAL with As_4_O_6_ in both parental HCT116 and HCT116-OxPt-R cells. This study demonstrated that As_4_O_6_ enhanced the anticancer effects of pKAL by inducing autophagic cell death accompanied by apoptosis in both parental HCT116 and HCT116-OxPt-R cells. In addition, it also suggests that the generation of ROS and the downregulation of AKT, NF-κB p65, cyclin D1, EGFR, and β-catenin may play an important role in the As_4_O_6_-enhanced anticancer effect of pKAL. However, the proteins that determine the anticancer efficacy of parental HCT116 cells and HCT116-OxPt-R cells appear to be different, suggesting that further research is needed on the differences in efficacy and the proteins that determine anticancer efficacy in other drug-resistant and radio-resistant cancer cells.

## Data Availability

Data is contained within the article.

## Abbreviations

OxPt	Oxaliplatin
OxPt-R	Oxaliplatin resistance
As_4_O_6_	Tetraarsenic hexoxide
pKAL	Polyphenols extracted from Korean *Artemisia annua* L.
LC3B	Microtubule-associated protein 1 light chain 3 beta
CD44	CD44 antigen
EGFR	Epidermal growth factor receptor
γ-H2AX	Phosphorylated form of histone H2AX on serine 139
PARP1	Poly(ADP-ribose) polymerase 1
PI3K	Phosphoinositide 3-kinase
ROS	Reactive oxygen species
NAC	N-acetyl cysteine
NRF2	Nuclear factor erythroid 2-related factor 2
As_2_O_3_	Arsenic trioxide
NF-κB	Nuclear factor kappa B
FDA	Food and Drug Administration
NT	Non-treated
ERK	Extracellular signal-regulated kinase
GAPDH	Glyceraldehyde-3-phosphate dehydrogenase
CCK-8	Cell counting kit-8
AKT	Protein kinase B (PKB)
SOD1	Superoxide dismutase [Cu-Zn]
Wort	Wortmannin
HO-1	Heme oxygenase-1
